# Diagnostic accuracy of Bethesda system for reporting thyroid cytopathology: an institutional perspective

**DOI:** 10.1186/1755-7682-7-46

**Published:** 2014-10-31

**Authors:** Samreen Naz, Atif Ali Hashmi, Amna khurshid, Naveen Faridi, Muhammad Muzzammil Edhi, Anwar Kamal, Mehmood Khan

**Affiliations:** Department of Histopathology, Liaquat National Hospital and Medical College, Karachi, Pakistan; Liaquat National Hospital and Medical College, Karachi, Pakistan; Dhaka Medical College, Dhaka, Bangladesh

**Keywords:** Bethesda system for reporting thyroid cytopathology (BSRTC), Fine needle aspiration cytology (FNAC), Thyroid nodule

## Abstract

**Introduction:**

Thyroid swelling is common problem among South Asian women. Although benign nodules far outnumber cancerous lesions, the risk of malignancy needs to be evaluated preoperatively for which fine needle aspiration cytology (FNAC) is widely used. Bethesda system for reporting thyroid cytopathology (BSRTC) was introduced to streamline the reporting of thyroid aspirates. We aimed to evaluate the disease spectrum of thyroid cytopathology and correlation of BSRTC with final histopathology in our setup.

**Methods:**

The study was conducted at Histopathology department of Liaquat National Hospital, Karachi, involving 528 patients with thyroid swelling who underwent FNAC. Out of these 528 cases, 61 patients subsequently underwent surgical excision. Results of final histopathology were correlated with cytologic diagnosis.

**Results:**

Mean age of the patients included in the study was 39.7 ± 13(14–84) and male to female ratio was 1:3.6. Out of total 528 cases, 403 cases were diagnosed as benign (Bethesda 2) and 67 were Bethesda 3 (follicular lesion of undetermined significance, FLUS) while 22 cases were categorized as either malignant or suspicious for malignancy (Bethesda 6 and 5). Histopathologic correlation was done in 61 cases. For Bethesda 5 and 6 categories, 100% concordance was found, however for Bethesda 2 category, 5 out of 45 cases were found to have malignant diagnosis on final histopathology. The incidence of malignancy in Bethesda categories 2 through 4 were 11.1%, 33.4%, 25%, 100% and 100% respectively. Overall accuracy of FNA cytology was 80.3% with 64.3% sensitivity and 85.1% specificity.

**Conclusion:**

Our study validated the accuracy of BSRTC in our setup. Therefore we recommend routine use of BSRTC for reporting thyroid cytopathology for initial workup of patients with thyroid nodule. However, risk of malignancy was found to be significantly high in Bethesda 3 category to warrant further workup including ultrasound/thyroid scan in addition to repeat FNAC.

## Introduction

Thyroid swelling is common problem among South Asian women due to probable dietary, environmental and genetic factors. Although benign nodules far outnumber cancerous lesions, the risk of malignancy needs to be evaluated preoperatively to determine the extent of surgery. Fine needle aspiration cytology (FNAC) was introduced for the same purpose and it soon gained wide acceptance among clinicians due to good patient compliance and cost effectiveness. As FNAC for thyroid swelling was found to have superior diagnostic reliability over ultrasonography and thyroid scintigraphy, American thyroid association and national comprehensive cancer network endorsed it as an initial diagnostic test of choice [1]. More over FNAC evaluation of thyroid nodules reduces load of unnecessary surgeries for benign lesion and opens the way to timely surgical intervention when there is significant risk of malignancy [2]. However, as some diagnoses cannot be reliably made on FNAC material like differentiation between follicular adenoma and minimally invasive follicular carcinoma, certain number of misdiagnosis are unavoidable. Inter-observer variability and inadequate aspiration are among some other limitations of this procedure.

Bethesda system for reporting thyroid cytopathology (BSRTC) streamlined the assessment and reporting of thyroid aspirates and alleviates the inter-observer variability of this procedure. BSRTC categorizes the FNAC diagnosis into six groups with well defined cancer risk and clear indications for further clinical management [3]. A few studies conducted in western countries reported a good diagnostic concordance between BSRTC and final histologic diagnosis; however such a data is scarcely available in our country [4]. There we aimed to evaluate the disease spectrum of thyroid cytopathology and correlation of BSRTC with final histopathology in our patient population.

## Methods

The study was conducted at Histopathology department of Liaquat National Hospital, Karachi. It involved 528 patients who presented with thyroid swelling and underwent FNAC. A 22 – 23 gauge needle was used for the procedure and smears and cell block preparation were made. Smears were stained with hematoxylin and eosin (H & E), giemsa and PAP stains. Repeat aspiration was done for cases with inadequate smears. FNAC were reported according to BSRTC by 2 experienced pathologists. Out of these 528 cases, 61 patients subsequently underwent surgical intervention, either excision of nodules/lobectomy or subtotal/near total thyroidectomy. Tissue specimens were grossly examined and processed according to standard guidelines and reported by senior histopathologists. Results of final histopathology were correlated with cytopathological diagnosis. Ethics committee of Liaquat National hospital approved the study.

## Results

Mean age of the patients included in the study was 39.7 ± 13(14–84) and male to female ratio was 1:3.6. Out of total 528 cases, 403 cases were diagnosed as benign (Bethesda 2) and 67 were Bethesda 3 while 22 cases were categorized as either malignant or suspicious for malignancy (Bethesda 6 and 5) as shown in Table 1. Histopathologic correlation was done in 61 cases which further underwent surgical intervention. For Bethesda 5 and 6 category, 100% concordance was found, however for Bethesda 2 category, 5 out of 45 cases were found to have malignant diagnosis on final histopathology. The incidence of malignancy in Bethesda categories 2 through 6 were 11.1%, 33.4%, 25%, 100% and 100% respectively (Table 2). Overall accuracy of FNA cytology was 80.3% with 64.3% sensitivity and 85.1% specificity (Table 3).

**Table 1:** **Cytologic diagnosis of 528 patients according to Bethesda System of Reporting Thyroid Cytopathology**

Bethesda category	Frequency	Percentage
Bethesda 1	25	4.7
Bethesda 2	403	76.3
Bethesda 3	67	12.7
Bethesda 4	11	2.1
Bethesda 5	18	3.4
Bethesda 6	4	0.8
Total	528	100.0

**Table 2:** **Correlation of Cytologic diagnosis with final histology, with incidence of malignancy in each Bethesda category**

Cytological diagnosis	Number of cases	Percentage	Histological diagnosis		Frequency	Percentage	Incidence of malignancy
Bethesda 2	45	73.77%	Benign	Nodular hyperplasia	6	13.3%	11.1%
				Multinodular hyperplasia	30	66.7%	
				Adenomatous hyperplasia	2	4.4%	
				Hashimoto thyroiditis	1	2.2%	
				Folicular adenoma	1	2.2%	
			Malignant	Follicular variant of papillary carcinoma	1	2.2%	
				Papillary carcinoma	3	6.7%	
				Medullary carcinoma	1	2.2%	
Bethesda 3	6	9.84%	Benign	Multinodular hyperplasia	3	50%	33.3%
				Adenomatous hyperplasia	1	16.7%	
			Malignant	Follicular variant of papillary carcinoma	1	16.7%	
				Follicular carcinoma	1	16.7%	
Bethesda 4	4	6.56%	Benign	Adenomatous hyperplasia	1	25%	25%
				Hashimoto thyroiditis	1	25%	
				Hurthle cell adenoma	1	25%	
			Malignant	Papillary carcinoma	1	25%	
Bethesda 5	4	6.56%	Malignant	Papillary carcinoma	3	75%	
				Non hodgkin lymphoma	1	25%	
							100%
		3.28%	Malignant	Papillary carcinoma	2	100%	
Bethesda 6	2						100%
Total	61	100.00%			61

**Table 3:** **Diagnostic accuracy of fine needle aspiration cytology according to Bethesda system of Reporting thyroid cytopathology**

True negative	40
False negative	5
False positive	7
True positive	9
Sensitivity	66.3%
Specificity	85.1%
Positive predictive value	56.3%
Negative predictive value	88.9%
Accuracy	80.3%

## Discussion

Thyroid diseases are one of the commonest health care problems in our population. Liaquat National Hospital is one of the largest tertiary care centers of the country with a large influx of patients from both the urban and rural parts of the province. Therefore our data is quite representative of entire population. Approximately 76% of thyroid swellings in our studied patients were benign on FNAC which only require surgical intervention for physical (pressure symptoms) or cosmetic reasons. Papillary carcinoma including its follicular variant represents the most common malignancy on final histology which is concordant with most of the national and international data [5].

Various researchers in different hospital and population based studies evaluated the diagnostic accuracy of thyroid cytopathology. Bagga et al., in a hospital based study in India involving only 32 cases found a diagnostic accuracy of 96.2% with 66% sensitivity and 100% specificity for FNAC thyroid. However they did not follow the Bethesda system and categorized results only into benign, suspicious for malignancy and malignant categories [6]. Gupta et al. evaluated 75 cases of solitary thyroid nodule. Histopathologic examination of excised specimens revealed 42 (56%) cases of colloid nodular goiter, 12 (16%) of follicular adenoma, 12 (16%) of papillary carcinoma and 3 (41%) of hurthle cell adenoma. Correlation of FNAC with histopathology revealed sensitivity, specificity, accuracy, false positive ratio and false negative ratio of 80%, 86.6%, 84%, 13.3% and 20% respectively [7].

In a Serbian study involving 266 patients with thyroid swelling, histopathologic correlation was done in 69 patents that later on underwent surgery. They found thyroid carcinoma in 10 patients with an overall accuracy of 97% [8]. Himakhm et al. in a hospital based study of 469 patients found malignancy in 179 cases, out of which 147 cases were that of papillary carcinoma. The sensitivity, specificity, positive predictive value, negative predictive value and accuracy were 82%, 100%, 100%, 90% and 93% respectively [9].

A few studies are also conducted in our population revealing spectrum of thyroid pathologies with cyto – histologic correlation. Mammon et al. in hospital based study in Islamabad involved 327 cases of thyroid FNACs, out of which 230 were categorized as benign, 64 suspicious for malignancy and 15 as malignant lesions. Subsequently 69 patients underwent thyroidectomy revealing an accuracy of 76.2%. Positive and negative predictive values were 50% and 94% respectively [10]. Another study conducted in Lahore compared results of 76 cases of FNAC with final histology. There were 30 benign lesions and 13 cases were malignant, the rest being categorized as indeterminate. They revealed an accuracy of FNAC to be of 87% [11]. Musani et.al in their group of patients assessed the role of FNAC in thyroid diseases. Out of 105 cases, 96 were benign and only 13 were malignant. They found a sensitivity and specificity of 61% and 99% respectively [12].

After the introduction of BSRTC, it was rapidly adopted by most institutions. Park et al. reported an incidence of malignancy for the six Bethesda categories to be 35.3%, 5.6%, 69.0%, 50.0%, 98.7%, and 98.9%, respectively [13]. In our study only 11% of cases in benign category were found to be malignant on final histology. Bukhari et. al compared the conventional reporting systems of thyroid cytopathology with newly introduced BSRTC and found a high diagnostic accuracy. They evaluated 120 specimens by three different reporting systems including BSRTC. There was no false negative diagnosis with 100% sensitivity compared to 77% and 85% sensitivity by conventional reporting systems. The specificity and positive predictive value was 82.5% and 45% respectively [14]. Similarly Al-Sindi et. al assessed 200 specimens according to BSRTC and found a sensitivity and specificity of 93% and 86% respectively. Only 6 cases in their series could be categorized as Bethesda 3 category, out of which 2 were malignant [15]. Bongiovanni et. al in a meta-analysis, compiled the results of 8 studies with 6362 cases having cyto-histologic correlation. They found sensitivity, specificity and diagnostic accuracy of 97%, 50.7% and 68.8% respectively. 9.6% cases were categorized as AUS/FLUS category and risk of malignancy was 15.9% [16].

Most of the studies conducted to date revealed a good accuracy of FNAC concordant with the results of our study, due to which it became a prime investigation of choice for initial evaluation of patients with thyroid nodule. However, literature review revealed that almost all the studies categorized their FNAC findings into three groups, benign, suspicious for malignancy and malignant with most of the patients falling in the second group. Unfortunately most patients undergoing surgeries with an initial diagnosis of suspicious for malignancy turned out be a benign lesion like adenomatous hyperplasia. Newly proposed Bethesda reporting guidelines categorized FNAC results of thyroid into 6 categorizes which eases the clinical intervention criteria and further reduces the chances of inadvertent surgeries.

In Bethesda system, only repeat FNAC is advised in follicular lesion of undetermined significance (FLUS) category. This diagnosis (Bethesda 3) is very important as cytologically unequivocal features of malignancy are absent, but few worrisome findings are present like focal nuclear enlargement/clearing or microfollicular pattern in a scanty smear which warrants repeat FNAC. We found that in Bethesda 3 category, 66.7% cases were benign while 33.3% turned out to be malignant. Rosario et. al in a study with 150 cases which were categorized as FLUS found a malignancy rate of 22.6% [17]. Gocun similarly reported a malignancy rate of 22.8% in FLUS/AUS category [18]. Our rate of malignancy was slightly high as compared to them; therefore we recommend a multidisciplinary approach to the management of cases diagnosed as FLUS including ultrasound, clinical workup and thyroid scan in addition to repeat FNAC.

One of the limitations of our study was that, only 10 out of 33 patients with cytologic diagnosis of Bethesda 4 through 6 categories underwent surgical intervention at our institution, the rest were operated at outside hospitals and therefore could not be followed. The high false positive and false negative rate in Bethesda 2 though 4 reflects incompetency of cytopathologists with Bethesda reporting at our institution and therefore require further training.

## Conclusion

We evaluated and reported our FNACs according to Bethesda guidelines and our study validated the accuracy of Bethesda system of reporting thyroid cytopathology in our setup. Therefore we recommend routine use of BSRTC for reporting thyroid cytopathology for initial workup of patients with thyroid nodule. However, risk of malignancy was found to be significantly high in Bethesda 3 category to warrant further workup including ultrasound/thyroid scan in addition to repeat FNAC.

### Consent

Written informed consent was obtained from the patient for publication of this manuscript and accompanying images. A copy of the written consent is available for review by the Editor-in-Chief of this journal.
